# Smoking habits and osteoporosis in community-dwelling men subjected to dual-X-ray absorptiometry: a cross-sectional study

**DOI:** 10.1007/s40618-024-02402-6

**Published:** 2024-05-28

**Authors:** A. Vergatti, V. Abate, L. D’Elia, G. De Filippo, G. Piccinocchi, L. Gennari, D. Merlotti, F. Galletti, P. Strazzullo, D. Rendina

**Affiliations:** 1 Department of Clinical Medicine and Surgery, Federico II University, Naples, Italy; 2 Assistance Publique—Hôpitaux de Paris, Hôpital Robert-Debré, Service d’Endocrinologie et Diabétologie, Paris, France; 3 COMEGEN” Medical Cooperative, Naples, Italy; 4 Department of Medicine, Surgery and Neurosciences, University of Siena, Siena, Italy; 5 Tobacco Treatment Center, Department of Clinical Medicine and Surgery, Federico II University, Naples, Italy

**Keywords:** Osteoporosis, Smoking habits, Cross-sectional study, Vitamin D

## Abstract

**Background:**

Active and Environmental Tobacco Smoke (ETS) are a global cause of death. Osteoporosis (Op) is the most common metabolic bone disorder worldwide, impacting on mortality and disability, with high health and welfare costs. Active smoking is a known risk factor for Op, but there is few information regarding Op and ETS in men.

**Purpose:**

The study aim is to evaluate the association between smoking habits and Op in community-dwelling men that have been subjected to Dual-X-ray Absorptiometry and completed a questionnaire about their own and cohabiter’s smoking habits.

**Methods:**

We performed a cross-sectional study based on administrative data. This study is part of the SIMON protocol. The binary logistic regression analysis was used to estimate the role of ETS on the risk of Op, adjusting for age, body mass index (BMI), type 2 diabetes mellitus (T2DM) and eGFR.

**Results:**

Four hundred sixteen men were selected and, based on questionnaire replies, 167 were classified as current smokers (CS), 93 as passive smokers (PS) and 156 as never smokers (NS). NS showed a lower prevalence of past fragility fracture, radiological features of osteoporosis and hypovitaminosis D compared to PS and CS (p < 0.05). NS showed a lower prevalence of Op compared to PS and CS, also after correction for age, BMI, T2DM and eGFR (p < 0.05).

**Conclusion:**

The study results demonstrate that PS and CS have a higher risk of Op, fragility fractures and vitamin D deficiency compared to NS.

**Supplementary Information:**

The online version contains supplementary material available at 10.1007/s40618-024-02402-6.

## Introduction

Active smoke and environmental tobacco smoke (ETS) are a main cause of death worldwide, leading to cardiovascular and lung diseases, cancer, and other debilitating conditions [1, 2]. The tobacco kills over 8 million people every year. In particular, about 7 million of deaths are caused by active smoke, while around 1.3 million are the result of exposure to ETS [3]. The World Health Organization (WHO) defines ETS as the combination of exhaled smoke from cigarettes and burning tobacco product [4]. ETS is especially found in homes, cars, and in public places, involving children above all [5]. The impact of ETS may be stronger than active smoke. It is estimated that the number of non-smokers exposed to ETS is on average higher than active smokers [6], increasing the burden on our health care system.

Osteoporosis (Op) is the most common metabolic bone disorder worldwide [7]. In 2020, 8.1% of the Italian population had a diagnosis of Op, with a higher prevalence in post-menopausal women and men older than 50 years [8]. Op is characterized by a pathological reduction in bone mineral density (BMD), assessed by Dual Energy X-Ray Absorptiometry (DXA), that increases the risk of fragility fractures [9]. These latter impact on mortality and disability, with high health and welfare costs [10].

Active smoking is a risk factor for Op and fragility fractures. Indeed, in Italy, according by essential levels of assistance (EALs, In Italian *Livelli essenziali di assistenza*) guaranteed by the Italian Ministry of Health, subjects that smoke more than 20 cigarettes/day must receive a DXA examination [11]. Available data regarding the relationship between ETS and Op are poor and mainly referred to women [12]. Few information is available regarding the Op risk in men exposed to ETS.

Therefore, based on administrative data, we performed this cross-sectional study to evaluate the association between Op and smoking habits in community-dwelling men, subjected to DXA according to Italian EALs.

## Methods

This study is part of the SIMON (in Italian *SIndrome Metabolica, Osteoporosi e Nefrolitiasi,* metabolic syndrome, osteoporosis, and nephrolithiasis) protocol [12–15]

**Fig.1:**
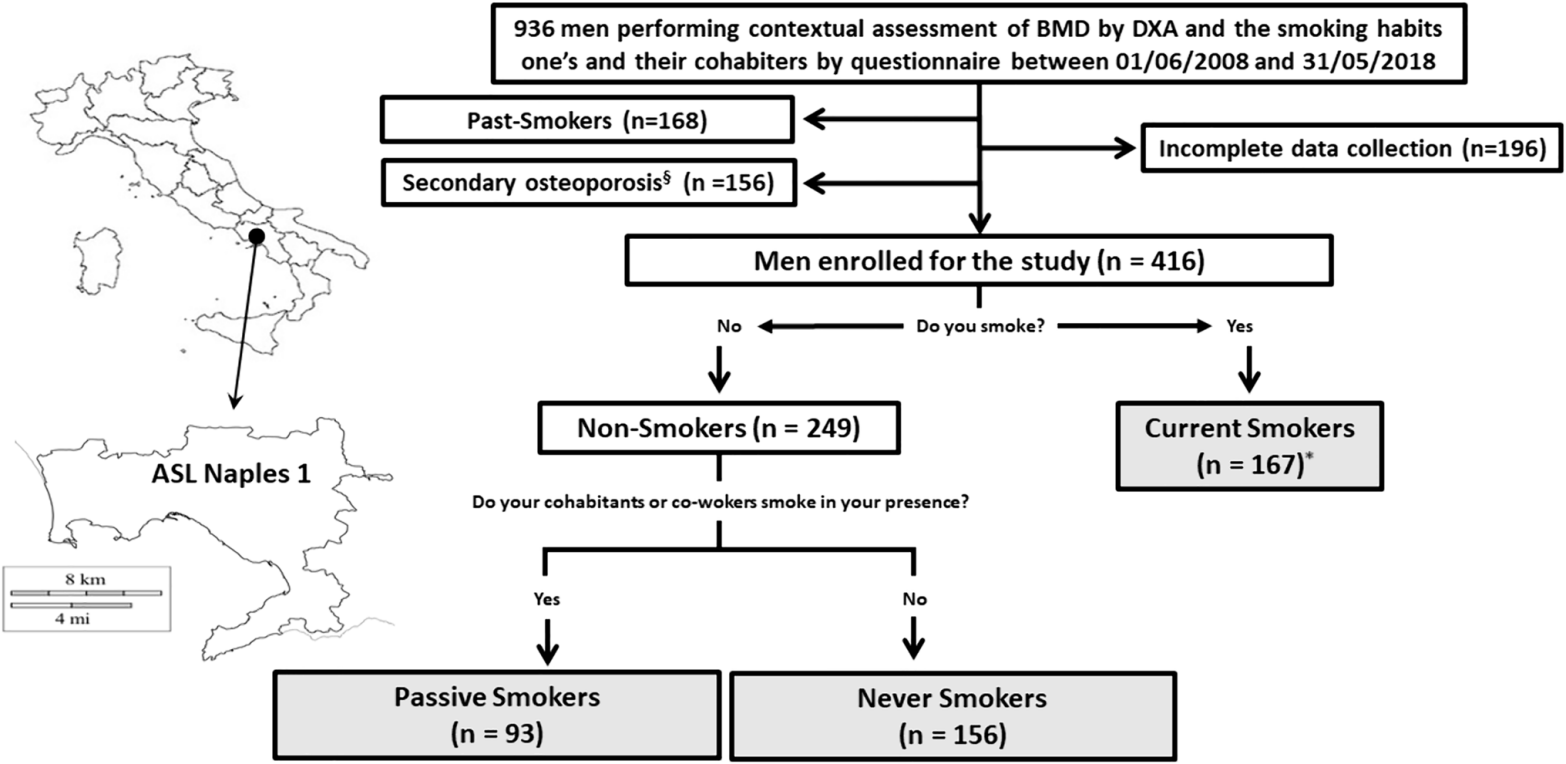
Study flowchart. The study populations are highlighted in grey. ASL Naples 1: Local Health Unit Naples 1. *BMD* bone mineral density evaluated by Dual-energy X-ray Absorptiometry. *DXA* Dual-energy X-ray Absorptiometry. §: subjects with malabsorption syndromes, rheumatoid arthritis, long-term immobilization, moderate to severe chronic kidney disease, hyperthyroidism, primary hyperparathyroidism, hypoparathyroidism, Cushing’s syndrome, chronic liver disease, pituitary tumours, surgical history of terminal ileal resection, gastrectomy or small bowel bypass, eating disorders, alcoholism, glucocorticoids, anticonvulsants, heparin, vitamin A and cytotoxic agents, were excluded from the study. * 107 CS smoke > 20 cigarette per day

The SIMON study is based on clinical data provided by general practitioners, belonging to the “COMEGEN” (in Italian *COoperativa di MEdicina GENerale*) Medical Cooperative, operating in the Local Health Unit (in Italian *Azienda Sanitaria Locale*, ASL) Naples 1 (Fig. 1). General practitioners selected men who had simultaneously undergone a DXA, according to EALs, and completed a questionnaire, about their own and their cohabiters’ smoking habits between June 1, 2008 and May 31, 2018.

The Op diagnosis was made based on DXA results (T-score value ≤  − 2.5 in the lumbar spine, total hip or femoral neck), according to the WHO diagnostic criteria [16]; and/or on radiographic images of fragility fractures; and/or on personal history of anti-osteoporotic treatment according to Italian Medicine Agency prescriptive criteria (in Italian, *Agenzia Italiana del FArmaco*—AIFA) [17].

The smoking habits were evaluated according to the following smoking questionnaire: (A) Have you ever smoked? Eventually, when did you start? (B) Have you ever smoked? Eventually, when did you stop? (C) Do your cohabitants or co-workers smoke in your presence? [12]. In case of positive answer to question A with cigarette consumption for at least 6 months, the subjects were classified as Current Smokers (CS). If the answer to question B was yes and the subjects had stopped smoking for at least 12 months, they were considered Past-Smokers and excluded from the study. If the answer to question C was yes, the subjects were defined as Passive Smokers (PS). Otherwise, they were defined as Never Smokers (NS) if the responses to question A, B and C were negative.

CS were additionally classified according to the number of cigarettes smoked per day: CS having less than or equal to 20 cigarettes per day and CS having more than 20 cigarettes per day. This latter is subjected to DXA prescription according to Italian EALs [11].

Clinical data regarding body mass index (BMI), age, sex, estimated glomerular filtration rate (eGFR, [18]), and pharmacological treatments were also collected.

The subjects younger than 40 years old and those with malabsorption syndromes, rheumatoid arthritis, long-term immobilization, estimated glomerular filtration rate lower than 60 ml/min/1.73 m^2^ [18], hyperthyroidism, primary hyperparathyroidism, hypoparathyroidism, Cushing’s syndrome, chronic liver disease, pituitary tumours, eating disorders, regular use of gonadotropin-releasing hormone agonist, glucocorticoids, anticonvulsants, heparin, vitamin A, cytotoxic agents for a current or past diagnosis of cancer, and history of Op treatment non-compliant to AIFA prescriptive criteria were excluded [15].

### Statistical analysis

Statistical analysis was performed using IBM SPSS (Statistical Package for Social Science), version 28 (IBM, Armonk, NY, USA). All variables showed a normal distribution to Kolmogorov–Smirnov test. In univariate analyses, statistical comparisons were based on the Student’s t test for continuous variables and on the Pearson’s Chi-squared test for dichotomous variables. The binary logistic regression analysis was used to estimate the role of ETS on the risk of Op, adjusting for age, BMI and eGFR. The collinearity among variables included in the models was assessed. The analysis did not detect any collinearity among variables (tolerance: 0.94, variance inflation factor 1.0). The results are reported as mean ± standard deviation (SD), or absolute, or percentages or as odds ratio (OR) and 95% confidence interval (95% CI). A p-value lower than 0.05 was considered significant.

The SIMON study was approved by the ASL Naples 1 Ethical Committee (protocol number 0018508/2018) and all enrolled subjects signed the informed consent.

## Results

As showed in Fig. 1, 936 community-dwelling men were subjected to DXA, and completed the questionnaire regarding their own and their cohabiters’ smoking habits. Four hundred sixteen of them [44.4%, mean age 68.86 ± 12.72 years; BMI 26.38 ± 3.63 kg/m^2^; eGFR 77.96 ± 3.63 ml/min/1.73m^2^] were selected, according to inclusion and exclusion criteria previously exposed. All selected subjects were older than 50 years. Based on smoking questionnaire replies, 167 men were classified as CS, 93 as PS and 156 as NS. Among CS, 107 (64.1%) were subjected to DXA because smokers of more than 20 cigarettes per day, as reported in Table 1.

**Table 1:** EAL criteria for BMD assessment in study population classified according to smoking habits and exposure to environmental smoke

	Non-Smokers		CS (n = 167)
EAL criteria	PS (n = 93)	NS (n = 156)	
Major risk factor for men of any age			
Previous frailty fractures	17 (18.3)	5 (3.2)^AB^	38 (22.7)
Radiological features of osteoporosis	63 (67.7)	62 (39.7)^AB^	95 (56.8)
Chronic therapies at risk of osteoporosis	0	0	0
Diseases at risk of osteoporosis	0	0	0
Minor risk factors for men > 60 years			
Age > 65 years	71 (76.3)	107 (68.6)	116 (69.5)
Family history of severe osteoporosis	45 (48.4)	70 (44.9)	61 (36.5)
Inadequate calcium intake	30 (32.2)	30 (19.2)	42 (25.1)
Vitamin D deficiency*	63 (67.7)	62 (39.7)^AB^	101 (60.5)
> 20 cigarettes/day	0	0	107 (64.1)
Alcohol abuse (> 60 g/day)	15 (16.1)	20 (12.8)	18 (10.8)

Data are expressed as absolute number (percentage). *EAL* essential assistance levels. *BMD* bone mineral density assessed by MOC-DXA. *Vitamin D deficiency is defined as vitamin D levels lower than 50 nmol/L [39]; among enrolled subjects with vitamin D deficiency 202 were treated with cholecalciferol and 22 with calcifediol. A significantly different compared to exposed (Chi-square test; p < 0.05); B significantly different compared to current smokers (Chi-square test; p < 0.05)

The criteria for DXA prescription according to EALs in the enrolled men are showed in Table 1. At the time of the examination, NS showed a lower prevalence of past fragility fracture, radiological features of Op and vitamin D deficiency compared to PS and CS (p < 0.05), respectively. Among enrolled subjects, 198 (47.6%) were treated with cholecalciferol and 22 (5.3%) calcifediol.

In the study population, 136 (32.7%) enrolled subjects were affected by Type 2 Diabetes Mellitus (T2DM). Of these, 84 have Op. Regarding smoking habits of T2DM men, 31 were CS, 53 PS, and 52 NS and the prevalence of diabetic subjects among CS, PS, and NS was not different.

**Fig.2:**
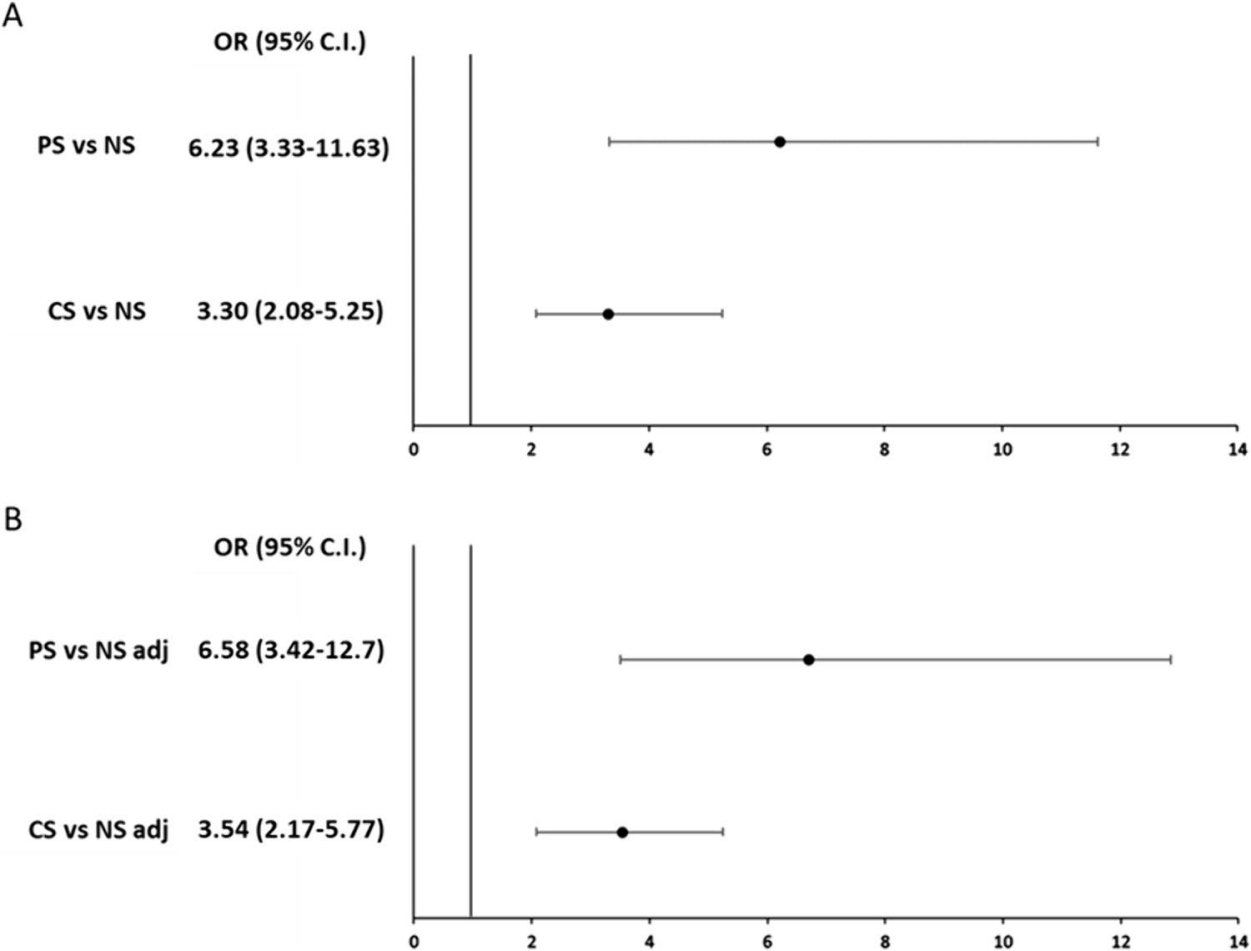
**A** Unadjusted Risk of osteoporosis in CS (current smokers), PS (passive smokers) and NS (never smokers) men enrolled in the SIMON study. **B** Risk of osteoporosis in CS, PS and NS men adjusted for age, body mass index (BMI) and estimated glomerular filtration rate (eGFR). *OR* odds ratio; *95% C.I* 95% confidence intervals, *adj* adjusted model

The overall prevalence of Op in enrolled subjects was 266 (63.9%). At univariate analysis, as reported in Table 2, the NS showed a lower Op prevalence compared to PS and CS (p < 0.05). Moreover, the prevalence of Op between CS > 20 cigarettes per day and CS ≤ 20 cigarette per day was not significant (73.8% vs 68.3%; p = 0.48). In the logistic regression analysis, the Op prevalence in PS was significantly higher compared to NS (Fig. 2a), also after correction for age, BMI, T2DM and eGFR (Fig. 2b). Similarly, the Op prevalence in CS was significantly higher compared to NS (Fig. 2a), after correction for age, BMI, T2DM and eGFR (Fig. 2b). No difference was found comparing the Op prevalence between CS and PS [OR 1.88 (1.00–3.56)].

**Table 2:** Clinical characteristics in study population classified according to smoking habits and exposure to environmental smoke

	Non smokers		CS
	PS	NS	
Number	93	156	167
Mean Age (years)	70.1 ± 12.9	68.6 ± 12.8	68.4 ± 12.6
BMI (Kg/m^2^)	26.7 ± 3.6	26.2 ± 3.7	26.3 ± 3.6
eGFR (ml/min/1.73m^2^)	79.8 ± 12.1	77.2 ± 10.4	77.6 ± 11.6
Osteoporosis	77 (82.8)	69 (44.2)^A,B^	120 (71.8)

Data are expressed as mean ± standard deviation and absolute number (percentage) for continuous or dichotomic variables respectively. *BMI* body mass index. A significantly different compared to passive smokers (Chi-square test; p < 0.05); B significantly different compared to current smokers (Chi-square test; p < 0.05)

## Discussion

The study demonstrates that PS and CS have an equal and higher risk of Op, fragility fractures and vitamin D deficiency compared to NS. The results were obtained in men, an often-neglected population from an osteo-metabolic perspective.

In a healthy condition, bone is featured by a continuous remodelling process, through osteoblast-mediated bone formation and osteoclast-mediated bone reabsorption [19]. Tobacco toxins impact on this dynamic process, impairing osteogenesis and/or increasing bone reabsorption, indirectly and directly [20].

Smoking indirectly impacts on bone health, altering BMI, PTH-vitamin D axis, adrenal and gonadal hormones, and increasing of oxidative stress. Nicotine, its metabolite, cotinine, and other smoke toxins cause a reduction in body weight, but they also act as endocrine disruptor, altering the balance between pituitary, adrenal and gonadal glands. It is demonstrated that smoking women experience menopause two years earlier than non-smoker women [21].

Moreover, nicotine has a direct effect on BMD inducing osteoblast apoptosis in vitro [22]. Furthermore, the Receptor Activator of Nuclear Factor-Kappa B Ligand (RANKL)—Receptor Activator of Nuclear Factor-Kappa B (RANK)—Osteoprotegerin (OPG) pathway play a key role in osteoclast differentiation, proliferation and activity [7]. Osteoblasts produce both RANKL, that stimulates osteoclast differentiation, and OPG, that inhibits osteoclast proliferation preventing the interaction RANKL-RANK [7]. Animal studies showed that rats exposed to smoke had higher RANKL/OPG ratio compared to rats not exposed to smoke [23, 24]. Human studies also confirmed these results in subjects exposed to smoke compared to non-exposed [25, 26].

CS and PS are not exposed to the same kind and amount of smoke. CS suffer with damage caused by mainstream smoke during a puff, and, on the other hand, PS inhale the smoke by burning tobacco products and the one exhaled by CS [27]. In addition, both CS and PS are exposed to the third-hand smoke, represented by the pollution resulting from a burning cigarette [28]. In confirmation of above, PS show higher serum, urine, and saliva levels of cotinine, as biomarker for smoke exposure, compared to CS [28].

Our study populations showed a higher prevalence of hypovitaminosis D in CS and PS compared to NS. This association is probably linked to the endocrine-disrupting role of tobacco smoke, impairing vitamin D intake, synthesis, hydroxylation, and catabolism [29]. Vitamin D is a fat-soluble hormone involved mainly in the maintenance of bone health. Vitamin D3 is synthesized in the skin and activated in the liver and then in the kidney, through a process of 25- and of 1-hydroxylation, respectively [29]. The final step is represented by 24-hydroxylation, that produces the vitamin D inactive metabolite.

Tobacco smoke acts on vitamin D synthesis, damaging the skin health through an increase in inflammation and aging processes [30]. The metal contained in tobacco causes renal damage, decreasing the activity of 1-alpha-hydroxylase [31], needed to vitamin D activation. Finally, smoking impairs the vitamin D catabolism, increasing the expression of cytochrome P450 24A1 (CYP24A1) [32].

The higher prevalence of hypovitaminosis D in CS and PS could be also related to the inhibition of PTH. Indeed, some studies showed lower PTH levels in CS than in non-smokers [33], linked to a direct activity of smoke both on calcium metabolism and parathyroid glands [34].

The relationship between Op, fragility fracture risk, T2DM and DXA is fascinating. The conventional World Health Organization criteria for Op diagnosis based on DXA data underestimate the risk of fragility fractures in patients with T2MD, showing a discrepancy between BMD and fracture risk [35]. Indeed, T2DM impairs bone quality, requiring ad hoc criteria for Op treatment [36]. In the SIMON population, we have already assessed the relationship between metabolic syndrome (hyperglycaemia/diabetes, hypertension, low HDL levels, hypertriglyceridaemia and high waist circumference) and osteoporosis [15]. In a logistic regression model including the individual components of metabolic syndrome, age, and ongoing treatments, a significant relationship between T2DM/hyperglycaemia and Op in men was not found [15]. Therefore, we have included T2MD subjects in our analysis.

The use of an administrative database represents both a strength and a limitation. It allowed to obtain a high number of enrolled subjects with low costs, and a highly sensitive and specific Op diagnosis based on DXA and medical history of use of anti-resorptive drugs [38]. The subjects underwent to DXA according to Italian EALs criteria. On the contrary, we have no data regarding the study population life style (physical activity, dietary habits, duration of exposure to ETS or exact number of cigarettes smoked per day, dietary calcium intake) or some biochemical and instrumental parameters (vitamin D levels and timing of its assessment, description of fractures and morphometric evaluation of vertebral fractures). Moreover, DXA results are obtained by different instruments, and our study population is not representative of all community-dwelling men, but only of subjects undergone to DXA.

The cross-sectional nature of the study prevents to establish a cause-effect relationship between ETS and Op, but to our knowledge, this is the first study regarding the association between Op and ETS in men undergone to DXA, an often-neglected population.

In conclusion, our study results suggest that in men PS have a higher risk of Op, fragility fractures and vitamin D deficiency compared to NS. CS and PS males present the same risk of Op, but further prospective studies are required to boost our results.

## Supplementary Information

Below is the link to the electronic supplementary material.

Supplementary file1 (DOCX 51 KB)

## Data Availability

The data that support the findings of this study are available on request from the corresponding author.

## Declarations

### Research involving human and animal participants

All actions carried out during this study were in accordance with the ethical standards of the institutional and/or national research committee and with the Helsinki Declaration and its amendments.
