# Supplementary material for: Smoking habits and osteoporosis in community-dwelling men subjected to dual-X-ray absorptiometry: a cross-sectional study
Source: J Endocrinol Invest. 2024 May 28;47(12):3129–35. doi: 10.1007/s40618-024-02402-6 (PMC11549200; doi:10.1007/s40618-024-02402-6)
Supplement: Supplementary file 1 — Supplementary file1 (DOCX 51 KB) [file 40618_2024_2402_MOESM1_ESM.docx]

Naples, May 15, 2024

Prof. Uberto Pagotto

Editors in Chief

Journal of Endocrinological Investigation

Manuscript n°: JENI-D-24-00347

Manuscript: Smoking habits and Osteoporosis in community-dwelling men subjected to dual-X-ray absorptiometry: a cross-sectional study.

Authors: Anita Vergatti, Veronica Abate, Lanfranco D’Elia, Gianpaolo De Filippo, Gaetano Piccinocchi, Luigi Gennari, Daniela Merlotti, Ferruccio Galletti, Pasquale Strazzullo, Domenico Rendina

Dear Editor,

Thank you for the attention you reserved to our work and for the possibility to resubmit it. We reviewed the manuscript taking into account the stimulating comments raised by the reviewers which allowed to significantly improve the manuscript.

All the issues raised by the referees have been carefully considered in the preparation of this revised version of our work.

On the bottom of the letter, you can find point-to-point answers to all reviewers’ issues.

All the authors of this revised version of the manuscript have consented to the changes made in the new version of the paper.

*COMMENTS TO THE AUTHOR:*

*Reviewer #1:*

*The work by Vergatti et al. investigates the relationship between osteoporosis and smoking in males, which is a population often neglected from an osteo-metabolic perspective, and notably passive smokers are included in the analysis.*

Thanks for your comments. We have added a sentence Discussion: “The study demonstrate that PS and CS have an equal and higher risk of Op, fragility fractures and vitamin D deficiency compared to NS. The results were obtained in men, an often-neglected population from an osteo-metabolic perspective”. (page 6, lines 129,130)

*The paper is interesting and may be considered for publication, but I'm asking for some major and minor revision:*

*Population screening:*

*1.The authors accurately ruled out numerous comorbidities that could be linked to secondary osteoporosis. However, diabetes mellitus, a well-known and very common cause of osteoporotic fractures, is not included in this list. Please add diabetes to the list of comorbidities if these data are available, or exclude patients with diabetes*

Thanks for your suggestion. As reported in the Material and Methods section, this study is a part of SIMON study protocol (page 4, lines 68-69). Indeed, a previous study [10.1007/s40618-021-01638-w] already evaluated the relationship between Op and metabolic syndrome and its constitutive components in the same study population (men subjected to DXA according to Italian EALs). In this setting, the study resulted demonstrated the lack of association between Type 2 diabetes mellitus and Op.

Based on your suggestion, we analysed the prevalence of type2 diabetes mellitus in Op subjects included in our study population: “In the study population, 136 (32.7%) enrolled subjects were affected by Type 2 Diabetes Mellitus (T2DM). Of these, 84 have Op. Regarding smoking habits of T2DM men, 31 were CS, 53 PS, and 52 NS and the prevalence of diabetic subjects among CS, PS, and NS was not different.” (page 6; lines 118-120)

Moreover, in the logistic regression analysis, we have adjusted the results also the occurrence of T2DM. “In the logistic regression analysis, the Op prevalence in PS was significantly higher compared to NS (Fig. 2a), also after correction for age, BMI, T2DM and eGFR (Fig. 2b). Similarly, the Op prevalence in CS was significantly higher compared to NS (Fig. 2a), after correction for age, BMI, T2DM and eGFR (Fig. 2b).” (page 6; lines 123-126)

We also added a sentence in the discussion section: “The relationship between Op, fragility fracture risk, T2DM and DXA is fascinating. The conventional World Health Organization criteria for Op diagnosis based on DXA data underestimate the risk of fragility fractures in patients with T2MD, showing a discrepancy between BMD and fracture risk [36]. Indeed, T2DM impairs bone quality, requiring *ad hoc* criteria for Op treatment [37]. In the SIMON population, we have already assessed the relationship between metabolic syndrome (hyperglycaemia/diabetes, hypertension, low HDL levels, hypertriglyceridaemia and high waist circumference) and osteoporosis [15]. In a logistic regression model including the individual components of metabolic syndrome, age, and ongoing treatments, a significant relationship between T2DM/hyperglycaemia and Op in men was not found [15]. Therefore, we have included T2MD subjects in our analysis.” (page 8; lines 163-170)

*2.The authors wrote that the patients younger than 40 years have been excluded from the study. However, the T-score is only applicable to males over the age of 50. Please rectify 40 with 50 if it was a typing error or exclude all the patients under the age of 50 years*

In the SIMON study protocol, based on administrative data and already exposed in previous studies [10.1007/s40618-021-01638-w, 10.1007/s40618-020-01428-w, 10.1007/s00223-020-00737-9, 10.1007/s40618-023-02211-3], we examined the clinical data of all subjects with age higher than 40 years referring to the general practitioners registered to the “COMEGEN” Medical Cooperative on June 1, 2018. However, all enrolled subjects in this study aged more than 50 years. In this regard, a sentence was added in the Results: “All selected subjects were older than 50 years old.”. (page 6; line 111)

*Methods:*

*1.Description of fragility fractures should be more detailed. Distinguishing major osteoporotic fractures like vertebral or hip fractures from other peripheral fractures would be appropriate*

*2.Please specify whether morphometric vertebral fractures have been included in the analysis, together with clinical fractures*

*3.Please clarify the method used to assess calcium intake and the timing of the blood test for 25OH-vitamin D in relation to enrolment*

*4.Specify whether the patients taking either cholecalciferol or calcifediol were excluded for vitamin D levels comparison*

Unfortunately, information regarding the fragility fractur description, the morphometric vertebral fractures, the method used to asses calcium intake and the timing of the blood test for vitamin D were not available, due to the nature of our study based on administrative data, as reported in the Introduction section of the manuscript (page 4, lines 65, 66). Indeed, we have added a sentence, recognizing these as study limitations: “On the contrary, we have no data regarding the study population life style (physical activity, dietary habits, duration of exposure to ETS or exact number of cigarettes smoked per day, dietary calcium intake) or some biochemical and instrumental parameters (vitamin D levels and timing of its assessment, description of fractures and morphometric evaluation of vertebral fractures)”. (page 8; lines 173-177).

Data regarding cholecalciferol and calcifediol supplementation in the study population are added in Table 1 caption: “204 (49.5%) subjects were treated with cholecalciferol and 22 (5.3%) calcifediol [17]”.

*5.Specify which threshold was used to define vitamin D sufficiency*

According to the Italian Society of Osteoporosis, Mineral Metabolism and Skeletal Diseases (SIOMMMS) guidelines, the vitamin D deficiency is defined by 25OHD levels lower than 50 nmol/L. In this regard a sentence was added to Table 1 caption: “*vitamin D deficiency is defined as vitamin D levels <50 nmol/L [40]”.

*6.Specify the mean vitamin D levels in the three groups*

We are sorry, but data regarding the mean vitamin D levels in the three-study population in not available, due to the nature of the study protocol. This additional limitation was reported in discussion section. (page 8, lines 175-177)

*Results:*

*1.If the meaning "radiological finding of osteoporosis" in Table 1 is related to DXA results, please consider to replace it maybe with "T-score <-2.5 SD" to avoid confusion with the term "osteoporosis" in Table 2, which I assume is related to both DXA T-score <2.5 SD and presence of fragility fractures*

We apologize for the lack of clarity in the data exposition. The data showed in Table 1 refer to the criteria by which general practitioners of COMEGEN cooperative prescribed DXA according to Italian EALs. Based on your suggestions, we replaced the words “radiological finding of osteoporosis” with the words “radiographic features of osteoporosis at plain radiograph” (https://radiopaedia.org/articles/osteoporosis-3#:~:text=with%20adult%20causes.-,Radiographic%20features,at%20for%20evidence%20of%20osteoporosis).

*2.Table 1 reports 100% score in CS for the outcome > 20 cigarettes per day. Does this mean that all current smokers were heavy smokers? Please provide further clarification on this conclusion as it is ambiguous. If only heavy smokers were included, it should be explicitly stated in the methods section*

We apologize for the mistake. CS that smoked more than 20 cigarette/day were 107 (64.1%). The other 60 CS undergone to DXA according to the EALs criteria not linked to smoking habits.

Based on your suggestion, some sentences were also added in the result section:

“Among CS, 107 (64.1%) were subjected to DXA, because smokers of more than 20 cigarettes per day, as reported in Table 1.” (page 6; lines 112,113);

“Moreover, the prevalence of Op between CS > 20 cigarettes per day and CS ≤20 cigarette per day was not significant (73.8% vs 68.3%; p=0.48).” (page 6, lines 122, 123)

*3.Figure 2 shows the results of both univariate and multivariate logistic regression analysis, but the presentation is unclear. Please consider separating the two analyses (for example place the univariate analysis at the top and the multivariate at the bottom).*

Thanks for the observation. The Figure 2 was modified, dividing the unadjusted and adjusted model.

*4.The odds ratio for current smokers compared to passive smokers is positive for CS in Figure 2, whereas the prevalence of osteoporosis is actually higher in passive smokers as stated in Table 2. Please consider correcting the results.*

Thanks for the comment. We have corrected the results and modified Figure 2:

“No difference was found comparing the Op prevalence between CS and PS [OR 1.88 (1.00-3.56)]” (page 6; lines 126, 127)

*Discussion:*

*1.This section is rather lengthy. Please shorten the pathophysiology section to focus solely on the impact of smoking on bone mass and vitamin D metabolism. I suggest minimizing the section comparing the present study with the SIMON study in women since the disparity in densitometry assessment between men and women is already well-established and it is not a focus of the present study.*

Thanks for the suggestion. Discussion was updated minimizing the comparison with the other studies belonged to the SIMON protocol, and focusing on the smoke effect on BMD and vitamin D metabolism.

*Reviewer #2:*

*This is an interesting manuscript because it provides evidence that passive smoking has a negative impact on bone and puts people at risk for osteoporosis defined by DXA.*

Thank you!!

*The authors need to have a native English speaker edit the manuscript or use an editing service to improve the English. There are many errors that must be corrected.*

We apologize for the grammatical mistakes. According to your suggestion a native English speaker edits the current version of the manuscript

*My major concern is with the findings illustrated in Figure 2. The risk of osteoporosis is about the same in current smokers and passive smokers. Yet, when compared to non-smokers, passive smokers are at much higher risk than current smokers compared to non-smokers. How is this possible? Please check your statistical analyses before re-submitting the manuscript.*

Thank you for the comment. The results were updated and a paragraph was added to the results and discussion, respectively:

“No difference was found comparing the Op prevalence between CS and PS [OR 1.88 (1.00-3.56)]” (page 6; line 126, 127)

“CS and PS are not exposed to the same kind and amount of smoke. CS suffer with damage caused by mainstream smoke during a puff, and, on the other hand, PS inhale the smoke by burning tobacco products and the one exhaled by CS [28]. In addition, both CS and PS are exposed to the third-hand smoke, represented by the pollution resulting from a burning cigarette [29]. In confirmation of above, PS show higher serum, urine, and saliva levels of cotinine, as biomarker for smoke exposure, compared to CS [29]”. (page 7; lines 145-149)

*Associate Editor*

*The English should be greatly improved and the statistical analyses carefully checked and revised accordingly.*

Thanks for the comment. The manuscript was improved and the statistical analyses was revised. In particular, we have adjusted the figure 2 according to the corrections, and two sentences were added in the result and discussion paragraphs respectively:

“No difference was found comparing the Op prevalence between CS and PS [OR 1.88 (1.00-3.56)]” (page 6; line 126, 127)

“CS and PS are not exposed to the same kind and amount of smoke. CS suffer with damage caused by mainstream smoke during a puff, and, on the other hand, PS inhale the smoke by burning tobacco products and the one exhaled by CS [28]. In addition, both CS and PS are exposed to the third-hand smoke, represented by the pollution resulting from a burning cigarette [29]. In confirmation of above, PS show higher serum, urine, and saliva levels of cotinine, as biomarker for smoke exposure, compared to CS [29]”. (page 7; lines 145-149)
